# Vision-Based Digital Twin and AI Agent Framework for Low-Cost, Explainable Indoor Building Inspection and Safety Assessment

**DOI:** 10.3390/s26154992

**Published:** 2026-08-06

**Authors:** Zijian Jing, Liyi Zhu, Tianyi Chen, Ludger Hovestadt, Li Li

**Affiliations:** 1 Key Laboratory of Urban and Architectural Heritage Conservation, School of Architecture, Southeast University, Nanjing 210096, China; 220230221@seu.edu.cn (Z.J.); e0348767@u.nus.edu (T.C.); 2 Group of Architects Co., Ltd. (GOA), 398 Tianmushan Road, Xihu District, Hangzhou 310058, China; zhu.liyi@goa.com.cn; 3 Institute of Technology in Architecture (ITA), 8093 Zurich, Switzerland; ludger@ethz.ch

**Keywords:** digital twin, AI agent, residential safety assessment, 3D reconstruction, existing housing stock, age-friendly environment

## Abstract

Aging residential buildings constructed under outdated design standards create an urgent need for scalable, evidence-based indoor safety assessment methods. Conventional manual inspections rely on subjective checklists, lack audit trails, and are impractical for widespread deployment. This study presents a vision-based digital twin and AI agent framework that converts a single continuous smartphone video into an explainable, evidence-constrained safety assessment. The pipeline employs MASt3R-SLAM to reconstruct a metric-scale 3D point cloud from monocular video, calibrated with AprilTag fiducials for absolute scale. SpatialLM parses the geometry to extract semantic entities and spatial relationships. Risk guidelines are formalized into a computable Risk Prototype structure, unified within a hierarchical SceneState data structure that binds geometric measurements, semantic labels, image observations, and regulatory knowledge. A LangGraph-based AI agent conducts a dual-pathway assessment: an initial whole-dwelling scan followed by iterative follow-up queries invoking tool calls for measurement, knowledge retrieval, or visual cross-checking. In a pilot validation across five heterogeneous residences, with detailed manual comparison in two representative cases, the framework achieved risk recall rates of 77.8–100% and precision rates of 45.0–70.0% against the single-assessor manual reference. The average judgment closure rate was 71.7%, with spatial granularity enhancement of up to 2.2× in complex environments. These results suggest that the framework can achieve risk coverage comparable to manual checklist inspection while offering enhanced granularity in complex environments and quantitative precision in well-defined spaces.

## 1. Introduction

A substantial proportion of the global residential building stock is now several decades old, and much of it was designed and constructed under standards that paid limited attention to the fine-grained spatial safety conditions of everyday domestic life. In China, rapid urbanization since the 1980s produced an immense inventory of residential units—apartment blocks, urban dwellings, and rural homes—that were built to meet acute housing shortages, with design priorities centered on basic shelter, floor-area efficiency, and construction speed rather than on occupant safety in daily activities such as ambulation, transfer, reaching, and bathing [1,2]. A significant share of this stock remains in active use today, yet its spatial configurations, dimensional relationships, and fixture installations were never systematically evaluated against the ergonomic and safety requirements of occupants across the life course [3]. As these buildings age, their inherent spatial deficiencies—narrow doorways, abrupt level changes, absence of support fixtures, inadequate illumination, and accumulations of household clutter in constrained circulation paths—compound into latent environmental hazards. The urgency of assessing and retrofitting this existing housing stock is magnified by the concurrent demographic reality of population aging: as of the end of 2024, the population aged 60 years and over in China reached 310.31 million, constituting 22.0% of the total national population, while those aged 65 and over numbered 220.23 million, or 15.6% [4]. Projections suggest that by 2050, the proportion aged 65 and above could exceed 26% [5]. National policy frameworks have explicitly endorsed a model of “home-based eldercare as the foundation, community-based support as the main support, and institutional care as a supplement” [6], which elevates the residential environment from a mere container of domestic life to a fundamental unit of the aging-in-place support system. The convergence of an aging building stock and an aging population thus creates a dual challenge: a vast inventory of homes whose latent spatial hazards were never designed to accommodate the diminished functional capacities of their elderly occupants.

The ecological theory of aging provides a robust conceptual framework for understanding why the spatial deficiencies of older housing stock become critical safety hazards for aging occupants. This theory posits that an individual’s functional ability is not solely a product of their intrinsic physiological and cognitive capacities but is co-determined by the interaction between these capacities and the demands of their surrounding environment [7]. As individuals age, they experience a progressive decline in key functional domains, including musculoskeletal strength, balance control, gait stability, visual acuity (particularly contrast sensitivity and dark adaptation), and reaction time [8]. These physiological changes effectively lower the threshold at which conventional spatial configurations and environmental features become hazardous. A residential environment that was once navigable and supportive can, in the context of diminished capacity, transform into a high-risk field for accidents. Empirical data underscores this vulnerability: over 70% of falls among older adults occur within the home or its immediate vicinity [9]. These incidents are rarely attributable to a single, isolated defect but are instead the result of a complex interplay between an individual’s declining abilities and specific environmental stressors. High-risk spatial units where this person-environment mismatch frequently manifests include thresholds and level changes, narrow circulation paths, wet areas like bathrooms and kitchens, and locations lacking adequate support for transfers and ambulation. Crucially, many of these hazardous spatial conditions are not random occurrences but are systematic features of housing built before age-friendly design principles were widely adopted, underscoring that the precise identification and characterization of environmental hazards in the existing building stock is not merely an architectural concern but a critical public health imperative, serving as the essential first step towards effective intervention and the restoration of a safe, supportive living environment.

Despite the clear need for systematic residential safety evaluation, current practices remain heavily reliant on manual, subjective methodologies that are fundamentally ill-suited to the scale and complexity of the challenge. The dominant approach involves trained professionals—such as occupational therapists, social workers, or building inspectors—conducting on-site visits to perform visual inspections against standardized checklists or screening tools like HOME FAST or HAP [10]. While these methods can be effective in controlled settings, they suffer from several critical limitations. First, they are highly dependent on the expertise, experience, and subjective judgment of the assessor, leading to potential inconsistencies in risk identification, severity grading, and problem attribution across different evaluators [11]. Second, they are resource-intensive, requiring significant time and personnel, which makes them prohibitively expensive and logistically challenging to deploy at the community or national scale necessary to address the burgeoning aging population, especially given the vast stock of existing, non-elderly-friendly housing [12]. Third, and perhaps most critically, the outputs of these assessments are often static snapshots in the form of a simple problem list or a scored checklist. They typically lack a stable, traceable organization of the underlying spatial evidence that led to each conclusion. This absence of an auditable evidence trail makes it difficult to verify findings, understand the specific conditions that constitute a risk, or support subsequent renovation planning and post-intervention verification [13].

Recent advances in artificial intelligence, particularly in multimodal large language models (MLLMs) and computer vision, have opened new avenues for automating aspects of environmental assessment. MLLMs have demonstrated impressive capabilities in scene description, object recognition, and visual question answering from 2D images [14]. However, a direct application of these general-purpose models to the rigorous task of residential safety assessment reveals significant and systemic shortcomings. Four primary limitations are evident. First, MLLMs operate primarily in a semantic space and lack an inherent, stable perception of true physical scale. They cannot reliably perform the quantitative measurements (e.g., doorway width, step height, clearance distance) that are often central to safety codes and risk determination. Second, their understanding is typically confined to local image patches and struggles to maintain a coherent, holistic representation of the 3D spatial topology of an entire dwelling, including the connectivity of rooms and the continuity of pathways across multiple viewpoints [15]. Third, they are not grounded in the specialized professional knowledge of building codes, accessibility standards, and gerontological principles that underpin expert risk judgment, limiting their ability to provide causal reasoning or cite authoritative sources for their conclusions. Fourth, their generative nature can lead to hallucinations or inconsistent outputs when prompted with slightly different phrasings or from different visual angles, undermining the reliability and reproducibility required for a safety-critical application [16]. Thus, while MLLMs offer a powerful foundation for visual understanding, they cannot, in their current form, serve as a standalone solution for a task that demands metric precision, spatial consistency, professional grounding, and evidential traceability.

The research landscape reveals a clear fragmentation between the domains of knowledge that are essential for a comprehensive residential safety assessment. On one hand, the fields of gerontology, architecture, and occupational therapy have produced a rich body of empirical knowledge, codified in design guidelines, assessment checklists, and regulatory standards [10]. This knowledge base effectively identifies common hazards and links them to fall outcomes, yet it remains largely trapped in natural language narratives or simple binary checklists, making it difficult to operationalize in a computational system. On the other hand, the fields of computer vision and robotics have developed sophisticated techniques for 3D reconstruction (e.g., SLAM, NeRF) and spatial semantic parsing (e.g., PointNet++, Segment Anything) that can objectively capture the geometry and object layout of an indoor environment. However, these technical outputs—point clouds, meshes, and object labels—are generic and not structured to answer the specific, context-dependent questions posed by safety guidelines. They provide raw data but not actionable, rule-based judgments.

The critical, unaddressed gap lies in the integration of these two worlds. There is a lack of a unified framework that can translate the qualitative, experience-based rules of safety assessment into a formal, executable structure that can directly query and reason over the quantitative, objective data provided by digital sensing technologies. While recent studies have demonstrated the efficacy of deep learning for automated, low-cost vision-based inspection of built environments—such as image-based building damage and crack detection in historical masonry structures [17]—these methods primarily focus on surface-level defect classification. Existing attempts to apply AI to indoor residential safety inspection often focus on either pure geometric analysis (e.g., detecting stairs or obstacles) or pure semantic labeling, without a mechanism to bind these observations to professional judgment criteria in a way that is both explainable and verifiable. Furthermore, there is no established method for organizing the heterogeneous evidence—spanning 3D geometry, 2D visual appearance, object semantics, and textual regulations—into a single, coherent factual record that can support a continuous, iterative process of hypothesis generation, evidence gathering, and conclusion refinement. This gap prevents the transition from a static, one-time inspection to a dynamic, intelligent diagnostic process that can adapt to user queries, fill in information gaps, and provide a transparent audit trail for every finding. The development of such an integrated, evidence-constrained framework is therefore a necessary and timely step to bridge the chasm between professional domain knowledge and advanced computational perception.

To bridge this critical gap, this study proposes a novel, integrated framework that synergistically combines low-cost vision-based sensing, structured knowledge representation, and an autonomous AI agent to create an explainable and evidence-driven residential safety assessment system. Our approach departs from prior work by establishing a formal pipeline that connects the physical world to professional judgment through a series of well-defined computational stages. The core innovation is the creation of a “digital twin” of the residence—not as a high-fidelity visual replica but as a structured, multi-modal factual database that serves as the substrate for intelligent reasoning.

**Figure 1:**
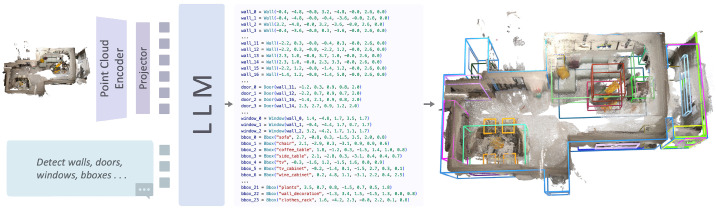
Representative Output of the SpatialLM Pipeline.

The framework begins with an exceptionally low-barrier data acquisition step: a single, continuous video recorded on an ordinary smartphone as the user walks through their home. This video serves as the sole input, reducing the barrier to data acquisition. From this unstructured video, we leverage the MASt3R-SLAM algorithm [18] to reconstruct a globally consistent, dense 3D point cloud that captures the geometric essence of the space. To overcome the inherent scale ambiguity of monocular vision, we introduce a simple AprilTag fiducial into the scene, which allows us to calibrate the reconstruction to absolute metric scale and establish a meaningful coordinate system. Concurrently, the SpatialLM model [19] processes this 3D geometry to extract a structured semantic skeleton, identifying and localizing key architectural elements (walls, doors, windows) and furniture objects, along with their spatial relationships (Figure 1).

The linchpin of our framework is the formalization of expert knowledge. We deconstruct traditional, narrative risk guidelines into a computable structure called the Risk Prototype. This prototype defines a risk not as a vague warning but as a precise set of five elements: the relevant behavioral context, the spatial scope, the specific judgment conditions, the required observable evidence, and the corresponding threshold basis. These prototypes are then used by an AI Agent, built on a LangGraph-based execution engine, to drive a continuous assessment process. The agent operates on a unified factual record, termed SceneState, which acts as a digital ledger that binds together all evidence modalities—geometric measurements, semantic labels, source video frames, and regulatory citations—around specific risk hypotheses. This architecture enables a dual-pathway workflow: an initial scan to build a comprehensive risk inventory, followed by an interactive, query-driven phase where the agent can invoke specialized tools for measurement, knowledge retrieval, or visual re-inspection when evidence gaps remain, thereby resolving uncertainties and providing detailed, evidence-backed answers.

This study aims to develop and preliminarily validate a vision-based digital twin and AI agent framework that restructures residential safety assessment into a continuous, evidence-constrained computational process. The specific research questions addressed are as follows: (1) How can empirical risk guidelines be formally decomposed into structured, computable information requirements? (2) How can heterogeneous sensing data be organized into a coherent factual record that supports risk verification? (3) How can an AI agent execution framework be designed to conduct continuous, tool-augmented assessment? (4) What is the preliminary performance of such a framework in diverse real-world residential environments compared to manual reference assessment?

The primary contributions of this work are methodological rather than algorithmic: we introduce (i) the Risk Prototype as a formalism for translating narrative risk guidelines into computable, evidence-constrained templates; (ii) the SceneState architecture for binding multi-modal evidence to structured risk hypotheses; and (iii) a dual-pathway agent workflow that separates initial whole-dwelling scanning from iterative, query-driven verification. The underlying technologies—MASt3R-SLAM, SpatialLM, LangGraph, and RAG—are existing open-source tools; our contribution lies in their task-specific orchestration and in the evidence organization principles that enable them to support safety-critical judgment.

The remainder of this paper is organized according to the following structure: Section 2, Methods, details the core components of our framework. It begins with the decomposition of risk mechanisms and information requirements, followed by a description of our geometric reconstruction and semantic parsing pipeline. It then introduces the SceneState architecture for evidence organization and concludes with the design of the AI agent’s orchestration and continuous assessment workflow. Section 3, Results, presents our experimental validation, including the characteristics of our residential samples, a detailed analysis of the system’s output and performance, and a comparative analysis with manual assessment. Section 4, Discussion, interprets these results, positions our methodology within the broader literature, acknowledges its limitations, and explores its practical implications. Finally, Section 5, Conclusion, summarizes the core contributions of this work, highlights its technical and practical significance, and outlines promising directions for future research.

## 2. Materials and Methods

### 2.1. Data Acquisition & Preprocessing

**Figure 2:**
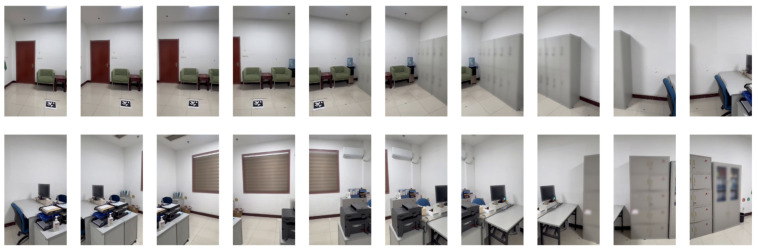
Representative Sequential Keyframes from Continuous Video Recording.

The foundational data for the residential environment age-friendly risk assessment framework is a single, continuous monocular video sequence captured using a standard smartphone. This acquisition protocol was deliberately chosen to ensure low deployment barriers and compatibility with real-world, non-controlled home environments. The recording procedure follows a “one-shot” scanning methodology: the user walks through the primary activity zones of the residence—including entryways, circulation paths, living rooms, bedrooms, kitchens, and bathrooms—while maintaining a steady, continuous motion without mid-process pauses or re-shoots (Figure 2). The camera is held at a natural human perspective, approximately at chest-to-head height, to ensure critical features such as thresholds, floor conditions, countertops, grab bars, and furniture clearances are within the field of view. To facilitate subsequent metric calibration, a physical AprilTag fiducial marker of known dimensions (tag family: 36h11; edge length: 0.165 m) is placed within the scene prior to recording. The AprilTag detection employs the apriltag Python library (Python 3.11.14; OpenCV 4.12.0 for tag generation, dictionary DICT_APRILTAG_36h11) library with default corner refinement parameters. Scale calibration solves for the optimal similarity transform $S^{*}\in \mathrm{Sim}\left(3\right)$ that aligns detected tag corners in the reconstructed point cloud with their known physical coordinates, averaged across multiple keyframe detections to reduce single-frame corner localization error. However, this calibration does not currently propagate formal uncertainty quantification: factors such as tag viewing angle, partial occlusion, corner detection sub-pixel error, and the similarity transform’s uniform scaling assumption may introduce scale errors that propagate to all subsequent measurements. A detailed uncertainty analysis of the calibration chain remains for future work.

This raw video input serves as the sole source for all downstream processing. The preprocessing pipeline begins by extracting a set of keyframes from the video stream, which are then fed into the MASt3R-SLAM system for 3D reconstruction. A critical preprocessing step for the reconstruction quality involves mitigating the adverse effects of specular reflections and glass surfaces, which are common in domestic settings and can severely degrade point cloud fidelity. In this preprocessing step, each frame undergoes a semantic pre-processing stage using a SegFormer model. This model generates a low-resolution semantic segmentation map, and the probability logits for target classes (e.g., windows, mirrors) are used to create a binary mask. Pixels within this mask are then subjected to Gaussian blurring, effectively suppressing their high-frequency texture content before they enter the geometric reconstruction pipeline. This operation reduces the influence of misleading visual cues on the cross-view matching process, leading to a more robust initial point cloud.

**Figure 3:**
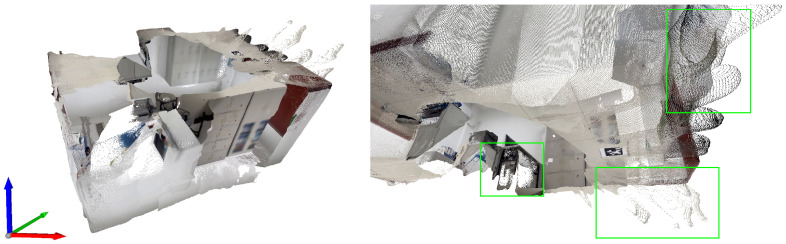
Typical Artifacts in the Point Cloud Output from MASt3R-SLAM. Green boxes highlight regions of low-quality point cloud reconstruction.

**Figure 4:**
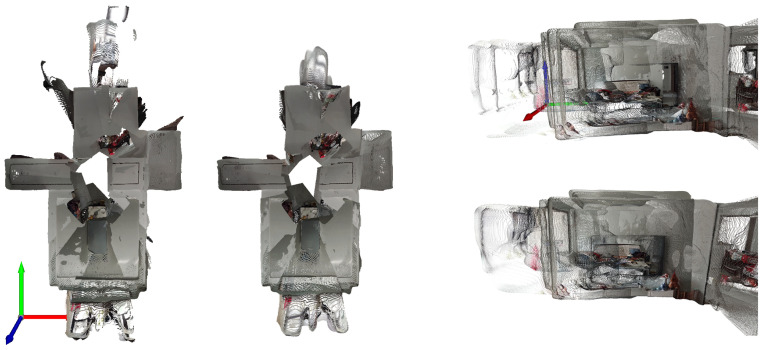
Comparison of Point Cloud Denoising Results Before and After Processing.

Following the generation of the raw point cloud and camera trajectory from MASt3R-SLAM, a post-reconstruction cleaning phase is applied. The original output, while dense, often contains artifacts such as floating noise points, linear streaks from fast camera motion, and sparse “spider-web” structures in occluded regions (Figure 3). To address this, a two-stage filtering process is employed. First, a quantile-based clipping operation is performed along the X, Y, and Z axes of the world coordinate system to remove extreme outliers that lie far outside the main spatial envelope of the residence. Second, a voxel-grid-based density filter is applied. The space is discretized into a 3D grid of voxels, and for each occupied voxel, its 26-connected neighborhood is analyzed. A voxel is only retained if its neighborhood meets a minimum occupancy threshold and contains a sufficient number of points. This step effectively removes isolated noise clusters and sparse, unreliable structures, resulting in a cleaner, more solid geometric base that is better suited for subsequent semantic parsing and measurement tasks (Figure 4).

**Figure 5:**
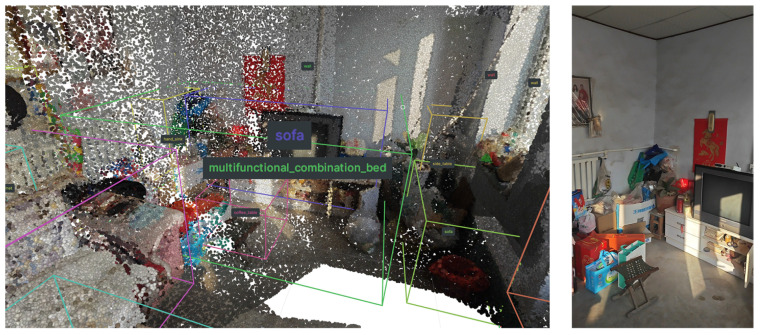
Representative Failure Cases in SpatialLM Recognition Results. Colored bounding boxes delineate different object categories.

Concurrently, the same keyframes used for geometry are processed by the SpatialLM model for semantic understanding. No specific image-level preprocessing is applied to these frames for the semantic pipeline, as the model is designed to handle naturalistic inputs. However, the quality of the semantic output is inherently dependent on the quality of the underlying point cloud, which has been enhanced by the aforementioned geometric preprocessing steps. The output of this stage is a set of structured semantic entities, including architectural elements (walls, doors, windows) and furniture objects (beds, chairs, cabinets), each annotated with its category, 3D position, scale, and orientation. This initial semantic skeleton, while useful, is treated as a hypothesis rather than ground truth, as it can contain errors due to occlusions, reconstruction artifacts, or model limitations (Figure 5). Therefore, a core part of the framework’s design is to allow this semantic state to be dynamically corrected during the agent’s execution phase through visual cross-checking against the original keyframes.

The specific parameters employed in the cleaning pipeline are as follows: quantile-based clipping retains points within the 1st to 99th percentile along each axis; the voxel grid size is set to 0.02 m; and a voxel is retained if at least 3 of its 26-connected neighbors are occupied and the total point count in the neighborhood exceeds 20. These parameters were empirically tuned on a small validation set of home scans to balance noise removal against preservation of thin structural elements.

### 2.2. Algorithmic Architecture/Mathematical Model

The core algorithmic architecture of this research is a multi-stage computational pipeline that integrates 3D reconstruction, semantic parsing, and an AI agent-driven reasoning engine, all unified under a novel evidence organization structure called SceneState. The mathematical foundation of the system rests on the formal decomposition of empirical risk guidelines into a computable structure—the Risk Prototype—and the precise definition of the geometric and semantic transformations that enable metric-scale verification from a monocular video.

**Figure 6:**
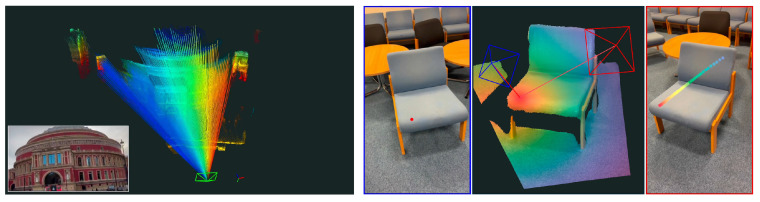
MASt3R Matching Enhancement Mechanism and Point Map Visualization. The conical wireframe indicates the camera frustum position.

The first major component is the 3D reconstruction module, which leverages the MASt3R-SLAM framework. At its heart, MASt3R-SLAM combines a learning-based dense geometry estimator (MASt3R) with a traditional SLAM back-end for global optimization. The MASt3R component, for a given pair of images $\left(I_{i},I_{j}\right)$, predicts a canonical point map $X_{\mathrm{canon}}\in R^{H\times W\times 3}$ for each image, where each pixel (u,v) is mapped to a 3D point $x_{u,v}$. It also outputs a dense descriptor map that facilitates robust cross-view matching even under challenging conditions such as occlusion or viewpoint changes (Figure 6). The SLAM front-end uses these predictions to estimate relative poses between consecutive frames, while the back-end builds a pose graph from keyframes and performs bundle adjustment to minimize reprojection error, yielding a globally consistent camera trajectory $\left\{T_{wc}^{k}\right\}$ and a fused point cloud P.

A critical mathematical transformation is then applied to recover absolute metric scale and a meaningful world coordinate system. The raw SLAM output is defined up to a similarity transform (rotation, translation, and uniform scaling). To resolve this ambiguity, an AprilTag of known physical size is used as a calibration fiducial. Let the four corner points of the detected AprilTag in the image plane be $\left\{p_{1},p_{2},p_{3},p_{4}\right\}$. These are projected onto the canonical point map $X_{\mathrm{canon}}$ of the corresponding keyframe to obtain their 3D coordinates in the camera frame, $\left\{x_{1}^{c},x_{2}^{c},x_{3}^{c},x_{4}^{c}\right\}$. Using the known camera pose $T_{wc}$ from SLAM, these are transformed into the SLAM world frame $x_{i}^{w}=T_{wc}\cdot x_{i}^{c}$. The known physical coordinates of the tag corners in its own local frame, $\left\{x_{1}^{\mathrm{tag}},\ldots ,x_{4}^{\mathrm{tag}}\right\}$, are then used to solve for the optimal similarity transform S∈Sim(3) that aligns the two sets of points:

$$S^{*}=\arg\;\min_{S}\sum_{i=1}^{4}{∥S\cdot x_{i}^{\mathrm{tag}}-x_{i}^{w}∥}^{2}.$$

**Figure 7:**
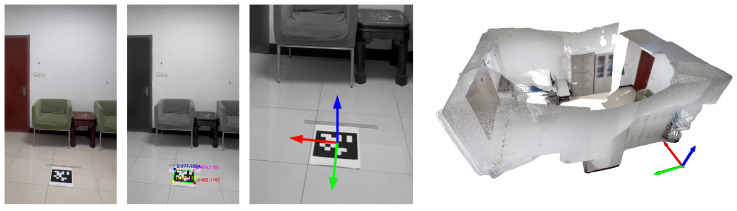
Schematic Illustration of Scale and Orientation Calibration Anchored by AprilTag.

This transform $S^{*}$ is then applied to the entire point cloud P and all camera poses, converting them from the arbitrary SLAM coordinate system into a physically meaningful one with meter-scale units and an orthogonal basis aligned with the tag’s axes (Figure 7).

For clarity, the principal mathematical symbols used in this section are summarized as follows: $I_{i},I_{j}$ denote input RGB images; $X_{\mathrm{canon}}\in R^{H\times W\times 3}$ denotes the canonical point map predicted by MASt3R, where each pixel (u,v) maps to a 3D point $x_{u,v}$ in camera coordinates; $T_{wc}^{k}\in SE\left(3\right)$ denotes the camera-to-world rigid transformation (pose) for keyframe *k*; P denotes the fused global point cloud; $\left\{p_{1},p_{2},p_{3},p_{4}\right\}$ denote the 2D image coordinates of AprilTag corners; $\left\{x_{i}^{c}\right\}$ and $\left\{x_{i}^{w}\right\}$ denotes the corresponding 3D corner coordinates in camera and world frames, respectively; $\left\{x_{i}^{\mathrm{tag}}\right\}$ denote the known physical corner coordinates in the tag’s local frame; and $S^{*}\in \mathrm{Sim}\left(3\right)$ denotes the optimal similarity transform (rotation, translation, and uniform scale) recovered by least-squares alignment.

The second major component is the evidence organization and reasoning engine. The fundamental unit of reasoning is the Risk Prototype, a structured template that decomposes a risk item into five core elements: (1) Behavioral Context (e.g., “transferring from bed”), (2) Spatial Scope (e.g., “a 1m radius around the bed”), (3) Judgment Conditions (e.g., “clear floor space must be ≥0.8 m wide”), (4) Observable Evidence (e.g., “floor area free of obstacles”), and (5) Threshold Basis (e.g., “GB 50763-2012 [20] §3.8.2”). This formalization allows the system to move beyond simple object detection to a conditional, context-aware verification process.

All evidence supporting or refuting a risk prototype is organized within the hierarchical SceneState data structure. Formally, SceneState is a tree-like data object with four primary layers:

1. Project Layer: Contains metadata for the entire assessment task (e.g., run_id, input video path).
2. Space Layer: Aggregates information per room or functional zone (e.g., “living room”), storing a summary of objects, identified risks, and open questions.
3. Frame Layer: Holds per-keyframe data, including the image path, camera pose $T_{wc}$, the canonical point map $X_{\mathrm{canon}}$, and any local observations or measurements made from that frame.
4. Object/Item Layer: Maintains a registry of all hypothesized objects with their properties (class, pose, scale) and a list of risk items, each linked to its evidence chain.

**Figure 8:**
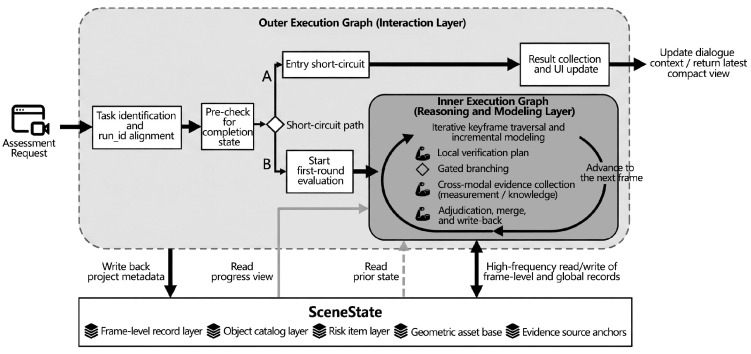
Nested Structure of the Dual-Layer Execution Graph.

The AI agent’s workflow is orchestrated as a state machine over this SceneState. The agent operates in two primary modes: an Initial Scan and a Follow-up Verification. During the initial scan, the agent processes keyframes sequentially. For each frame, it queries the current SceneState to build a compressed context, then uses a multimodal LLM to perform a local assessment. This assessment involves generating a set of risk hypotheses, identifying potential object misclassifications, and proposing necessary tool calls (e.g., for measurement or knowledge lookup). A gating mechanism then decides the next action based on the presence of evidence gaps. This process is formally represented as a directed acyclic graph (DAG) using LangGraph, where nodes are processing functions (e.g., frame_eval, measure, adjudicate) and edges represent state transitions conditioned on the output of the previous node (Figure 8).

The follow-up verification mode is triggered by a user query. The agent first parses the query to determine its intent (e.g., “MEASURE”, “RISK_EXPLAIN”) and its spatial scope. It then navigates the SceneState hierarchy to locate the relevant information. If the existing state is insufficient to answer the query, the agent formulates a plan to invoke the necessary tools. For instance, to measure a doorway width, the agent would first select the most suitable keyframe(s) where the doorway is visible, then generate a computer vision program to detect the door jambs in the 2D image, and finally use the pre-computed $X_{\mathrm{canon}}$ and $T_{wc}$ to lift these 2D points into the metric 3D world and compute the Euclidean distance between them.

### 2.3. Implementation & Tuning

The implementation of the proposed framework is a sophisticated integration of several open-source libraries and custom-built modules, designed to bridge the gap between high-level AI reasoning and low-level geometric computation. The core execution environment is built upon Python (version 3.11.14), leveraging PyTorch (version 2.4.1+cu124) for deep learning models and a suite of specialized libraries for 3D processing and agent orchestration.

Recent advances in open-source multimodal LLMs and agent frameworks have made this integration feasible. The Qwen3-VL series [21] provides the visual-language reasoning backbone, while the LangChain and LangGraph frameworks [22,23] supply the tool-calling abstractions and cyclic execution control necessary for long-horizon assessment tasks. For 3D reconstruction, MASt3R-SLAM [18] builds upon the DUSt3R dense matching paradigm [24], which has demonstrated robust cross-view correspondence under challenging indoor conditions. SpatialLM [19] extends this geometric foundation with structured semantic parsing, drawing on the broader trend of 3D scene understanding via large language models [14]. The RAG pipeline employs Chroma [25] for vector storage and OpenCLIP [26] for cross-modal retrieval, reflecting established practices in retrieval-augmented generation for knowledge-intensive tasks [27].

The 3D reconstruction pipeline is implemented using the official MASt3R and MASt3R-SLAM codebases. Key implementation details include the use of a fixed keyframe selection interval during the SLAM process to balance reconstruction density with computational cost. The AprilTag detection and pose estimation are handled by the apriltag Python library. The similarity transform calibration is implemented using a least-squares solver from the scipy.optimize module. The point cloud cleaning pipeline utilizes the Open3D library for voxel grid creation and neighborhood analysis, with the occupancy threshold and minimum point count per neighborhood being tuned empirically on a small validation set of home scans to maximize the removal of noise while preserving thin structural elements like chair legs or door frames.

The semantic parsing component relies on the SpatialLM model, which is loaded and executed using the Hugging Face transformers library. Its input is the cleaned point cloud from the reconstruction stage, formatted as an array of XYZRGB points. The output is a JSON-formatted list of detected objects and architectural elements. This initial semantic output is directly ingested into the SceneState’s object registry as a set of hypotheses, each marked with a source_state of “spatiallm_initial”.

The AI agent’s reasoning engine is powered by the Qwen3-VL family of multimodal large language models (specifically, the Qwen3-VL-Plus and Qwen3-VL-Flash variants), accessed via API. To balance computational efficiency and diagnostic depth, a dual-model strategy is employed: high-frequency, lightweight tasks (e.g., initial image filtering and fast semantic extraction) are routed to Qwen3-VL-Flash, while complex adjudications and multi-step tool calling are handled by Qwen3-VL-Plus. The agent’s workflow is a complex, stateful program managed by the LangChain and LangGraph frameworks, where the entire agent logic is encoded as a state graph with the SceneState object as its state.

**Figure 9:**
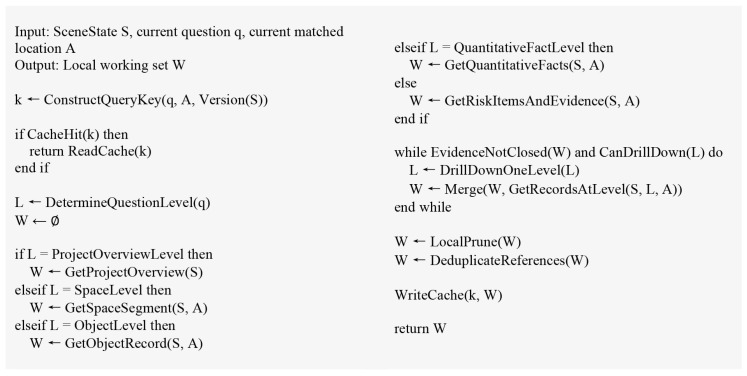
Pseudocode Illustration of the Compressed View Assembly Workflow.

**Figure 10:**
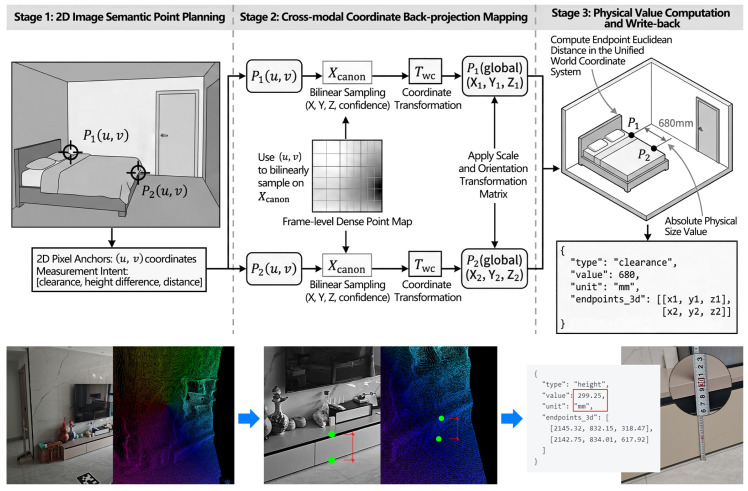
Principle and Workflow of the Geometric Measurement Pipeline. The colored point cloud facilitates spatial visualization. In the middle panel, green dots mark the measurement endpoints, and the red line indicates the measured dimension. In the right panel, the red box highlights the measurement result.

**Figure 11:**
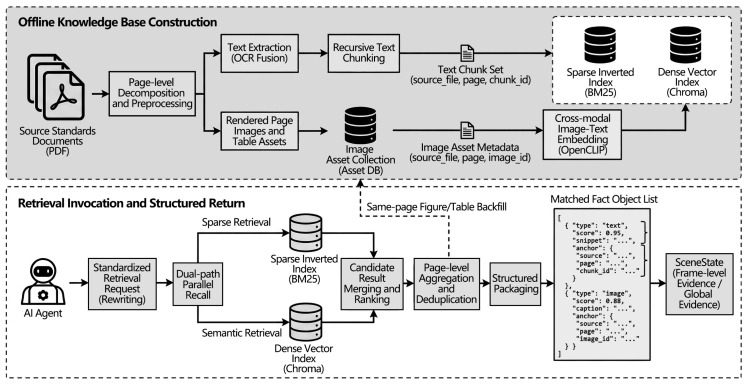
Implementation Framework of the Knowledge Supplementation Tool.

A significant implementation effort went into designing the tool suite that the agent can call. Four primary tool types were engineered:

1. Record Query: This tool interfaces directly with the SceneState JSON file, allowing the LLM to fetch specific slices of information (e.g., “get all objects in the kitchen”) without having to process the entire state. It implements a hierarchical querying mechanism with caching to prevent redundant file reads (Figure 9).
2. Image Review: This tool loads a specific keyframe image and allows the LLM to request a re-evaluation of a particular region or object class, facilitating runtime correction of the initial SpatialLM output.
3. Geometric Measurement: This is perhaps the most complex tool. It accepts a measurement type (e.g., clear_width_between_boundaries), a target object, and a frame ID. It then uses the LLM to generate a Python script that uses OpenCV to detect the relevant features (e.g., door jambs) in the 2D image. The 2D pixel coordinates are then mapped to 3D world coordinates using the frame’s $X_{\mathrm{canon}}$ and $T_{wc}$, and the final metric distance is computed (Figure 10).The geometric measurement tool generates Python code for 2D feature detection using OpenCV. To mitigate execution risks, the generated code is run within a sandboxed environment with restricted imports and no network access. The tool implements failure detection through the following: (i) validation that detected 2D features fall within expected semantic regions (e.g., door jambs must lie within wall-door transitions); (ii) confidence scoring based on feature detection stability across multiple candidate points; and (iii) geometric plausibility checks (e.g., computed widths must be positive and within physically reasonable bounds for residential spaces). Failed measurements are logged with diagnostic information and retained as open evidence gaps rather than propagated as confirmed values. Nevertheless, the comprehensive validation of the LLM-generated code against a ground-truth annotated dataset and the formal verification of the sandbox containment remain incomplete and are identified as priorities for future work.
4. Knowledge Retrieval (RAG): A dedicated offline pipeline was built to construct a vector database of relevant regulatory documents, including national standards like GB 50096 [28] and local guidelines like DB4403/T 399-2023 [29]. This pipeline uses Chroma for vector storage, BM25 for sparse keyword retrieval, and OpenCLIP for cross-modal (text-to-image) retrieval of diagrams and tables. At query time, the RAG tool performs a hybrid search and returns a structured list of relevant text snippets and image assets with their source citations (Figure 11).

Prompt engineering is a critical aspect of the implementation, with a layered strategy to constrain the LLM’s behavior at different stages of the workflow. For example, the prompt for the frame_eval node strictly limits the LLM’s output to a predefined JSON schema containing fields for observed objects, risk hypotheses, and proposed tool calls. This prevents the LLM from generating free-form text that would be difficult to parse programmatically. Similarly, prompts for the measurement tool instruct the LLM to output only valid Python code that conforms to a specific API for feature detection. These constraints are essential for maintaining the stability and reliability of the automated pipeline.

### 2.4. Validation Strategy

The validation strategy was designed as a pilot study to preliminarily assess the framework’s performance in realistic, heterogeneous home environments. The validation has several constraints: the sample size is small (N = 5), the manual comparison involves only two samples, the manual baseline employs a single assessor without inter-rater reliability testing, and no ground-truth tape measurements were systematically collected. The results should therefore be interpreted as indicative of feasibility rather than as definitive evidence of generalizability.

The pilot dataset consisted of five real-world residential units, selected to represent a broad spectrum of housing conditions in China: a newly built urban apartment (Sample 01), a rural dwelling with non-standard construction (Sample 02), an early-2000s urban home occupied by a mobility-impaired elder (Sample 03), and two typical 1980s-era urban apartments (Samples 04 and 05). This heterogeneity was intended to stress-test the system’s robustness, not to support statistical generalization.

Samples 02 and 04 were selected for manual comparison because they represented, respectively, a spatially complex rural dwelling and a typical aged urban apartment. The remaining three samples (01, 03, 05) were reserved for system-level testing across diverse conditions but were not included in the manual comparison due to assessor availability constraints. This is a recognized limitation.

**Figure 12:**
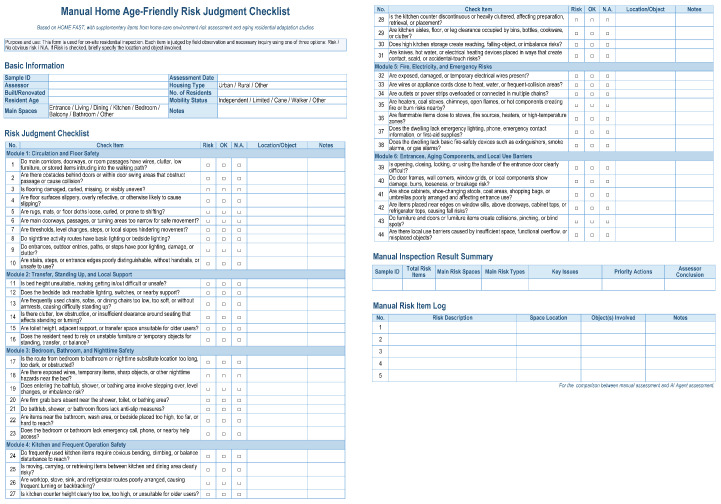
Manual Assessment Checklist used for structured on-site observation.

For the two comparison samples, the following procedures were conducted:

1. Agent-Based Assessment: A single continuous video was recorded following the prescribed protocol. The video was processed through the full pipeline, and the system’s output—a structured report of risk items with evidence chains—was recorded. Targeted follow-up queries were posed to probe refinement capability.
2. Manual Assessment: A single assessor (the first author) conducted an on-site inspection using a comprehensive checklist adapted from the HOME FAST instrument [30] and localized Chinese guidelines (Figure 12). The assessor documented identified hazards, locations, and justifications through systematic observation and measurements. This manual assessment serves as the reference standard for this pilot comparison, providing the best available baseline against which the framework’s indicative performance can be characterized.

The comparative analysis focused on three dimensions:

- Coverage and Agreement: Overlap between risk sets identified by both methods. A “match” required both methods to flag the same hazard type in the same spatial location. The analysis cataloged complete matches, partial matches, manual-only findings (treated as false negatives relative to the reference), and agent-only findings (treated as false positives relative to the reference). Building on this matching taxonomy, derived performance indicators—recall, complete match rate, miss rate, and precision—were computed against the manual reference to provide indicative quantification of the framework’s relative performance. No statistical agreement metrics (e.g., Cohen’s kappa) were computed due to the single-assessor design.
- Granularity and Precision: The manual assessment typically produced room-level summaries (e.g., “kitchen has clutter issues”). The agent’s output was more granular, pinpointing specific objects and spatial relationships (e.g., “a stack of boxes on the fridge top creates a fall risk when reaching”). For quantitative thresholds (e.g., doorway width), the agent provided metric values through its geometric measurement tool, though these values have not been validated against independent tape-measure ground truth.
- Evidence Traceability: Qualitative assessment of whether risk conclusions could be traced to supporting evidence (keyframes, measurements, knowledge references) within SceneState. The manual checklist did not systematically document evidentiary basis in a structured format.

This pilot validation provides preliminary evidence that the framework can operate across diverse residential conditions and achieve coverage comparable to a single-assessor manual checklist in the two tested cases. It does not establish superiority, generalizability, or measurement accuracy.

#### Ablation Study Limitation

The current pilot design does not include ablation experiments that would isolate the contribution of individual pipeline components (MASt3R-SLAM, SpatialLM, RAG, SceneState, and the AI agent execution graph). Consequently, the incremental value of each module relative to the integrated whole cannot be quantified. For example, it remains unverified whether the dual-pathway workflow (initial scan plus follow-up verification) outperforms a single-pass approach, or whether the SceneState evidence structure improves risk identification accuracy over an unstructured prompt chain. Conducting controlled ablation studies—such as evaluating the system without SceneState, without RAG-based knowledge retrieval, without geometric measurement tools, or without the follow-up verification phase—is essential for future work to justify the architectural complexity of the full framework. These experiments were deferred due to the limited sample size and the absence of a ground-truth annotated dataset; they are identified as a priority for subsequent research.

## 3. Results

### 3.1. Experimental Setup and Sample Characteristics

**Figure 13:**
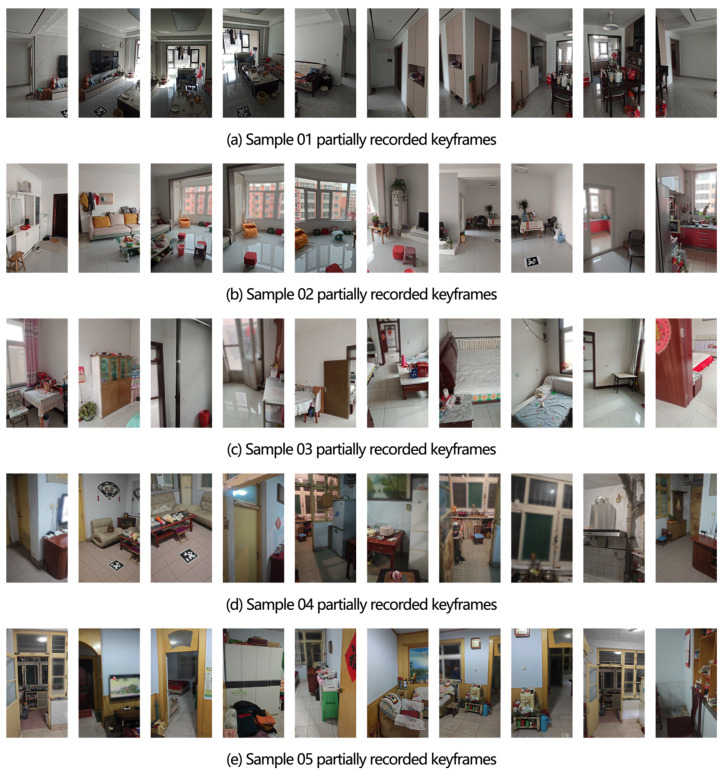
Representative Keyframes Collected from Each Sample.

The validation study employed the same five real-world residential units described in Section 2.4 (Figure 13). This selection strategy ensured that the system was tested against varying levels of spatial complexity, furniture arrangement, renovation period, and resident functional capacity.

Data collection for each sample followed a standardized protocol using a common smartphone. A single, continuous video scan was performed, capturing the primary activity spaces—including entrances, circulation paths, living rooms, kitchens, and bathrooms—without any mid-recording interruptions or supplemental close-up shots. This approach preserved the natural observational constraints of a real-world deployment, including transient occlusions, variable lighting, and brief camera movements, thereby providing a realistic testbed for the system’s robustness.

For comparative analysis, a manual assessment was conducted on Samples 02 and 04 by a trained assessor using the comprehensive checklist described in Section 2.4, covering six key modules: circulation and ground safety, transfer and support, bedroom/bathroom/night safety, kitchen safety, fire/electrical/emergency risks, and entrance/component aging issues. The performance of the AI agent framework was evaluated against this manual baseline across four primary metrics: overall risk coverage, the spatial granularity of findings, the explicitness of evidence traceability, and the rate of judgment closure versus open evidence gaps.

### 3.2. System Output and Risk Identification Performance

**Figure 14:**
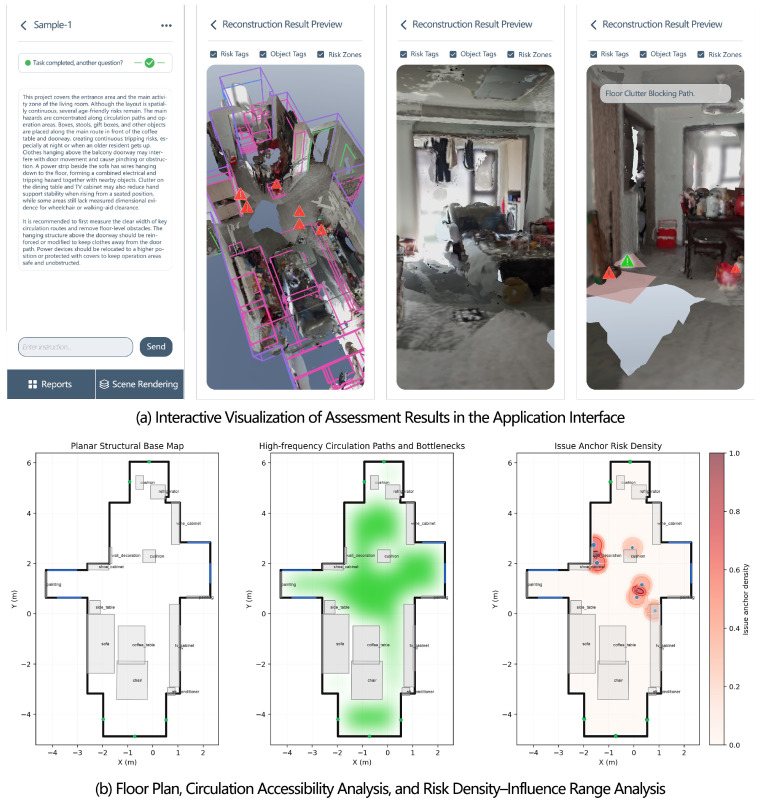
Overview of Assessment Results for Sample 01. In the 3D scene, colored bounding boxes indicate recognized object types: green for doors, purple for enclosing walls, magenta for furniture, and blue for windows. Red triangular markers denote risk locations; upon selection, these markers turn green and display a detailed risk description. In the floor plan, green shading represents feasibility analysis, with darker tones indicating higher occupant usage frequency; red zones mark risk locations and their influence ranges.

**Figure 15:**
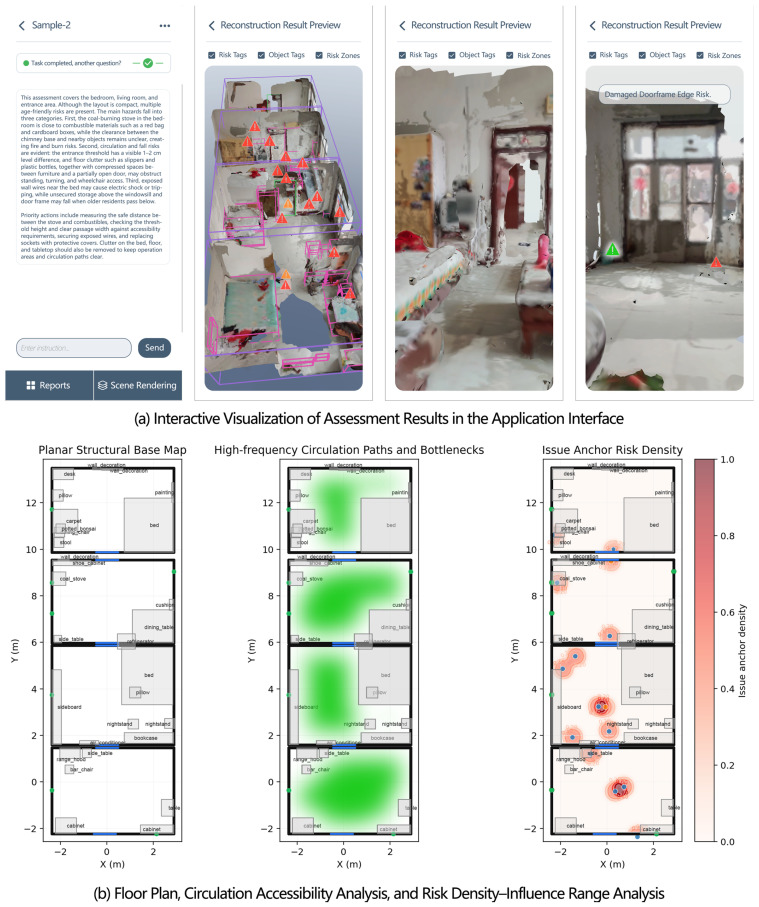
Overview of Assessment Results for Sample 02. In the 3D scene, colored bounding boxes indicate recognized object types: green for doors, purple for enclosing walls, magenta for furniture, and blue for windows. Red triangular markers denote risk locations; upon selection, these markers turn green and display a detailed risk description. In the floor plan, green shading represents feasibility analysis, with darker tones indicating higher occupant usage frequency; red zones mark risk locations and their influence ranges.

**Figure 16:**
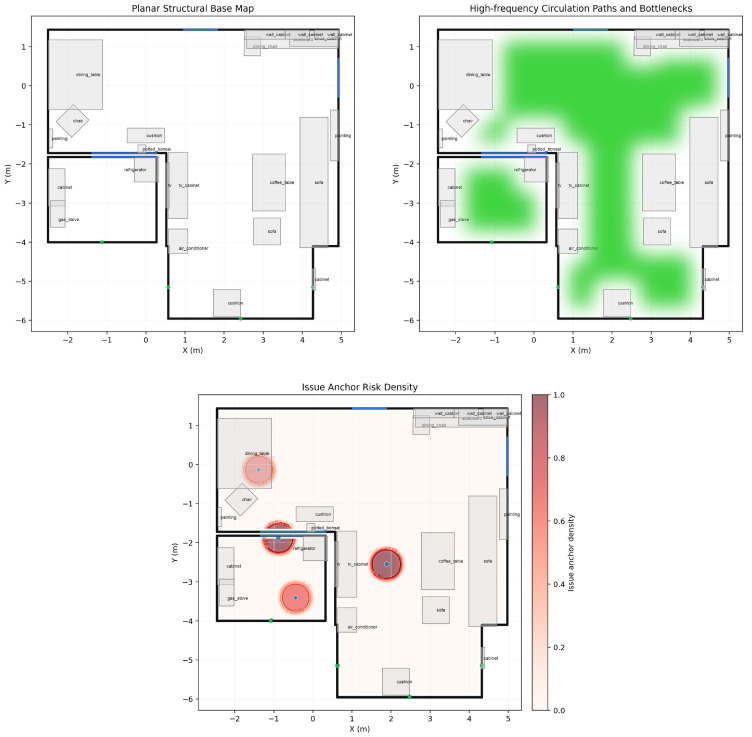
Floor Plan, Circulation Accessibility Analysis, and Risk Density–Influence Range Analysis Results for Sample 03. Green shading represents feasibility analysis, with darker tones indicating higher occupant usage frequency. Red zones mark risk locations and their influence ranges.

**Figure 17:**
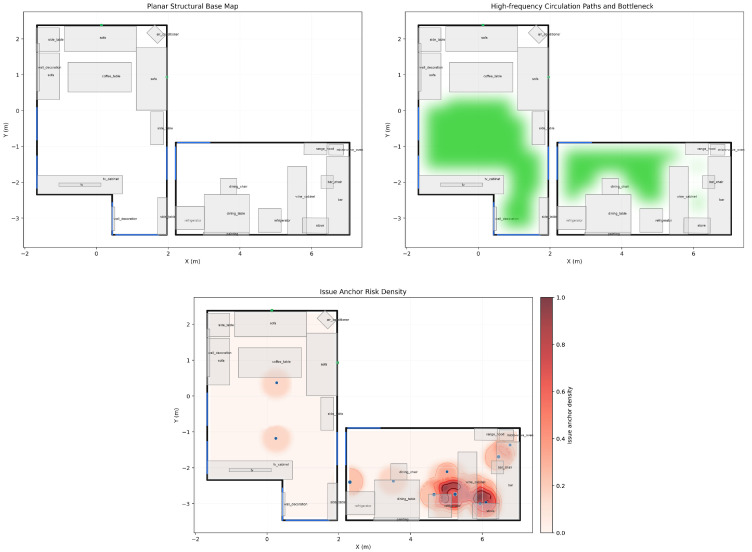
Floor Plan, Circulation Accessibility Analysis, and Risk Density–Influence Range Analysis Results for Sample 04. Green shading represents feasibility analysis, with darker tones indicating higher occupant usage frequency. Red zones mark risk locations and their influence ranges.

**Figure 18:**
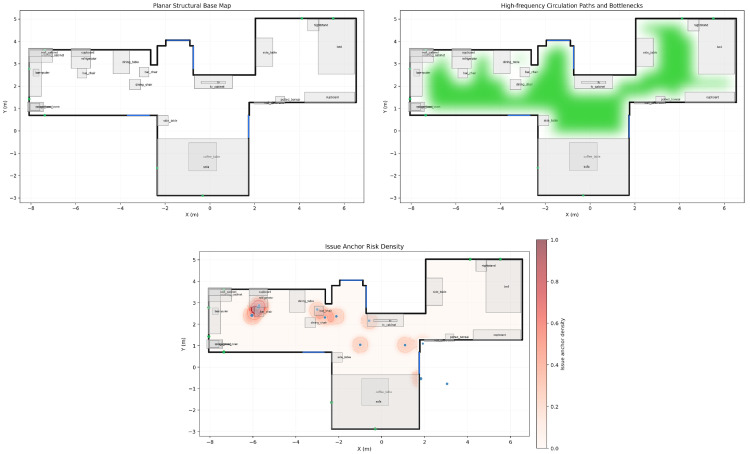
Floor Plan, Circulation Accessibility Analysis, and Risk Density–Influence Range Analysis Results for Sample 05. Green shading represents feasibility analysis, with darker tones indicating higher occupant usage frequency. Red zones mark risk locations and their influence ranges.

The AI agent framework successfully processed all five residential samples, generating a structured project output for each that included a textual report, a 2D analytical diagram, and a 3D model with back-projected risk annotations. The core of this output is the SceneState data structure, which organized findings into discrete risk entries. In the following overview (Figure 14, Figure 15, Figure 16, Figure 17 and Figure 18), the 2D diagrams and 3D models are deliberately designed as illustrative tools to provide visual spatial context for the identified risk items. They are not intended to convey quantitative accuracy themselves; all quantitative comparisons and performance metrics are reported in the accompanying text and in Table 1. We acknowledge that the visual density of these composite figures may affect readability, and we prioritize the textual and tabular results as the primary evidence for performance assessment. Each entry comprised a clear spatial localization, a causal explanation of the hazard, references to relevant regulatory knowledge, and a status flag indicating whether the judgment was fully closed or retained as an open evidence gap pending further verification.

In the new urban apartment (Sample 01), characterized by clear spatial boundaries and modern fixtures, the system identified six primary risk items. Five of these were fully closed, pinpointing hazards such as ground obstacles in the main circulation path and clutter around the TV cabinet that could impede movement. One item remained open, flagged for further verification of a local passage condition, demonstrating the system’s conservative approach to uncertain evidence (Figure 14).

The rural dwelling (Sample 02) presented a significantly more complex challenge, featuring a central corridor linking multiple functional zones, non-standard furniture (e.g., coal stoves, folding tables), and numerous temporary obstructions. Despite the high semantic ambiguity for the underlying SpatialLM parser, the agent’s continuous assessment logic proved robust. It generated 20 risk items, 14 of which were closed in the initial scan. These included highly specific hazards like the risk of tripping over loose wires beside the bed at night and the insufficient clearance when a door was opened into a protruding broom. Six items were retained as open gaps, primarily concerning precise measurements of object heights or clearances that were not fully observable in the initial pass (Figure 15).

For the residence of a mobility-impaired elder (Sample 03), the system demonstrated its capacity for adaptive risk profiling. Informed by the user-provided context of the resident’s need for a walking cane, the assessment prioritized low-height obstacles and support-related issues. It identified seven risk items, including a fish tank and scattered shoes in the entrance that posed a direct interference for a cane user, and a glass coffee table in the living room that offered no safe support during sit-to-stand transitions (Figure 16). This case illustrates how the framework can modulate its focus based on occupant-specific functional data, moving beyond a one-size-fits-all checklist.

The two aged urban apartments (Samples 04 and 05) yielded results that were both stable and representative of common issues in older housing stock. In Sample 04, the system identified 10 risk items, 7 of which were closed, covering a range of problems from deteriorated door frames and suboptimal kitchen counter heights to clutter on top of the refrigerator (Figure 17). Similarly, in Sample 05, 11 risk items were generated, with 7 closed, focusing on narrow doorways, blind spots in circulation, and accumulated clutter around key activity zones like the kitchen and TV area (Figure 18). Across all samples, the system consistently produced multi-layered visual outputs. The 2D analytical diagrams integrated a base floor plan with overlays for circulation paths, risk density, and evidence maturity, while the 3D models allowed users to visually inspect the exact location of each annotated risk within the reconstructed space.

### 3.3. Pilot Comparative Analysis with Manual Assessment

This section presents a pilot comparison between the agent-based assessment and the single-assessor manual inspection for Samples 02 and 04. Building on the matching taxonomy introduced in Section 3.3.1, we organize the analysis into five complementary dimensions: (1) definition of the reference standard and matching framework, (2) matching statistics between agent and manual outputs, (3) derived performance indicators computed against the manual reference, (4) judgment closure analysis across all five samples, and (5) spatial granularity enhancement.

#### 3.3.1. Reference Standard and Matching Framework

For the purpose of this pilot comparison, the manual assessment described in Section 2.4 serves as the reference standard (ground truth). Each risk item identified by either method was classified into one of four matching categories:

- Complete Match (CM): Both methods independently identified the same hazard type at the same spatial location with substantially equivalent descriptions.
- Partial Match (PM): Both methods identified related hazards at the same location but with differences in specificity, scope, or risk attribution.
- Manual-Only (MO): A risk item identified by the manual assessor but not captured by the agent, treated as a false negative relative to the reference.
- Agent-Only (AO): A risk item generated by the agent but not recorded in the manual assessment, treated as a false positive relative to the reference.

We note that this reference standard reflects the judgment of a single assessor rather than an independent gold standard; its role is to provide the best available comparison baseline for characterizing the framework’s indicative behavior.

#### 3.3.2. Matching Statistics

A direct item-level comparison was performed on the rural dwelling (Sample 02) and the aged urban apartment (Sample 04). Table 1 summarizes the matching statistics.

In the spatially complex rural dwelling (Sample 02), the manual assessment identified 9 primary risk areas, providing a useful high-level overview but lacking fine-grained detail. For instance, it noted “clutter by the bed” and “fire risk from stove” as general categories. In contrast, the AI agent decomposed these broad categories into 20 specific, actionable items. It distinguished between a hanging wire on the headboard, loose items on the bedside table, and a decorative object on the windowsill as separate trip hazards. For the stove, it specified the compressed emergency egress space due to adjacent cardboard boxes. Of the 20 agent-identified items, 7 were in complete agreement with the manual findings, 2 were partially aligned, and 11 were unique to the agent’s analysis. Notably, no risk category identified by the manual assessor was entirely missed by the agent (MO = 0). This demonstrates the agent’s stronger capacity for local problem decomposition in environments with high object density and ambiguous spatial boundaries (Table 1).

**Figure 19:**
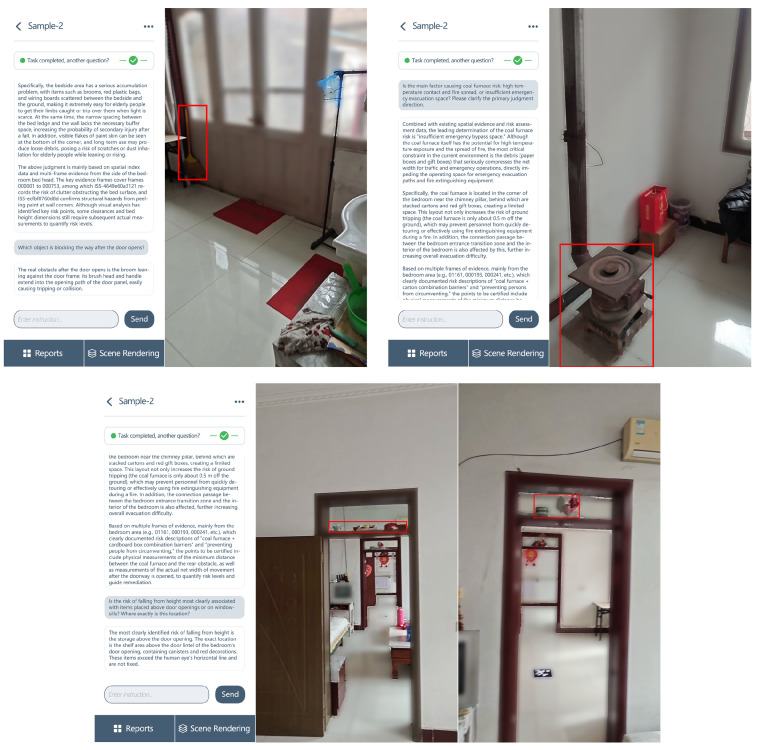
Agent Follow-up Assessment Results and Image Evidence Traceback for Sample 02. Red boxes highlight the specific regions identified as risk factors.

The follow-up questioning phase was particularly impactful in this sample. When prompted, the agent could refine its initial hypotheses, for example, by confirming that the primary nighttime risk at the bedside was a combination of a trailing power cord and a low stool, and by precisely locating the source of a potential falling-object hazard to a specific shelf above a doorway (Figure 19).

**Figure 20:**
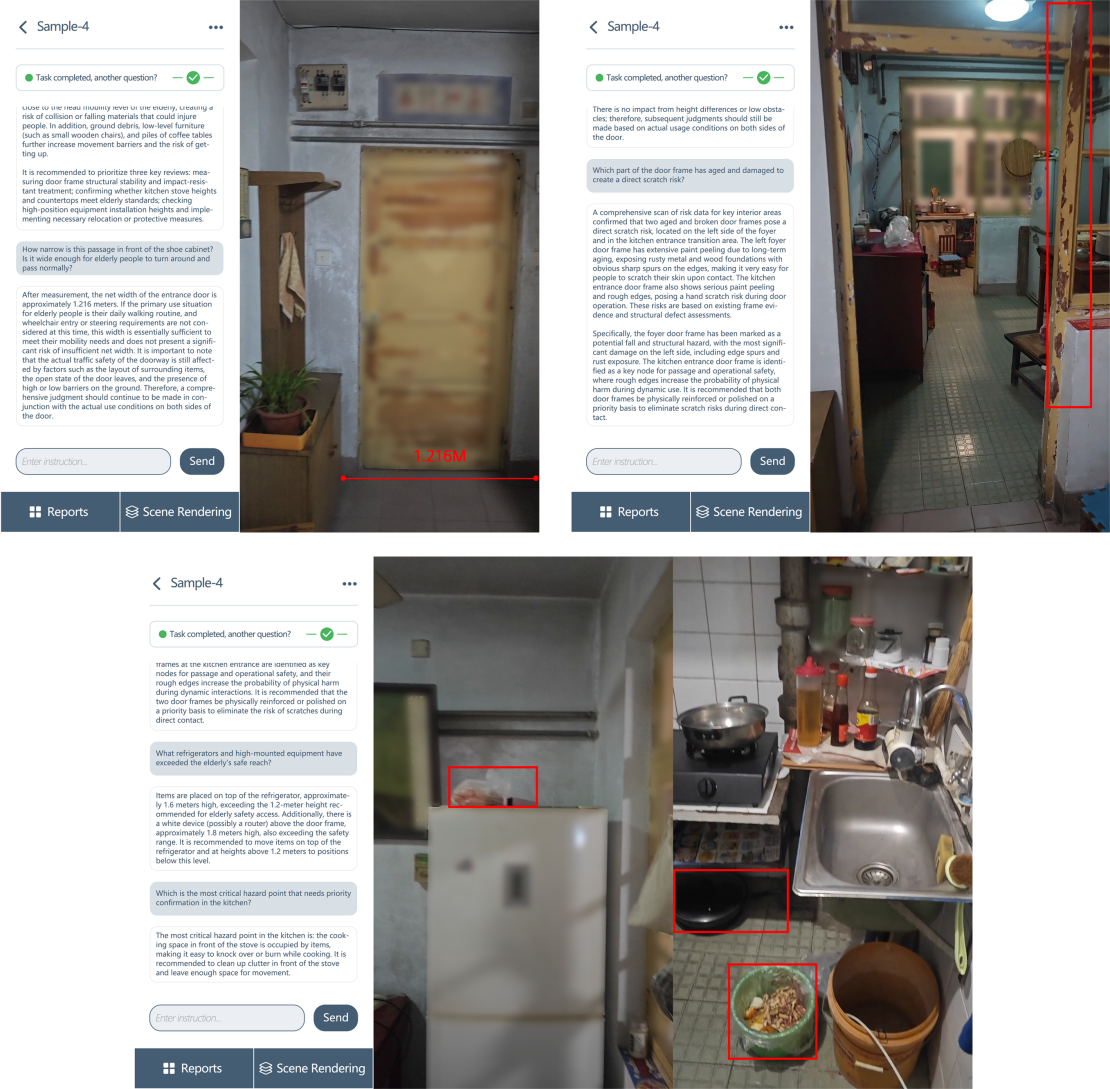
Agent Follow-up Assessment Results and Image Evidence Traceback for Sample 04. Red boxes highlight the specific regions identified as risk factors.

In the more structured aged urban apartment (Sample 04), the performance of the two methods converged (Table 1). The manual assessment identified 9 key issues, which were largely concentrated in the kitchen and entrance areas, such as a low kitchen counter, a soft sofa making it hard to stand, and an old, damaged door frame. The AI agent’s initial scan identified 10 items, showing strong alignment with the manual findings. Here, the agent’s primary advantage was not in discovering new risk categories but in providing enhanced precision and verifiability. For example, a manual note of “narrow entrance” was transformed by the agent into a quantified measurement of a 1.216 m doorway width after a targeted follow-up query. Similarly, the general observation of “deteriorated door frame” was refined to specify the exact locations of two damaged frames and their associated laceration risk (Figure 20). In this sample, 5 of the agent’s 10 items were in complete agreement with the manual assessment, 2 were partially aligned, 2 were unique to the manual method (MO = 2), and 3 were unique to the agent (AO = 3). The two manual-only items both concerned the absence of emergency call devices—a risk category that relies less on visual–spatial evidence and more on explicit equipment inventory, revealing a boundary condition of the visually driven framework.

#### 3.3.3. Derived Performance Indicators

From the matching statistics in Table 1, four indicative performance indicators were computed against the manual reference. Table 2 reports these indicators along with their definitions.

##### Recall

(R=77.8–100.0%, mean 88.9%). This indicator measures the proportion of manually identified risk categories that were also captured (completely or partially) by the agent. The maximum recall of 100% in Sample 02 indicates that, in this spatially complex environment, the agent did not miss any risk category that the manual assessor had flagged. The lower recall in Sample 04 (77.8%) is attributable to the two emergency-call-device items, which fall outside the framework’s visually driven evidence scope. These results suggest that, within its visual–spatial competence domain, the framework achieves risk coverage comparable to the single-assessor reference.

##### Complete Match Rate

($R_{\mathrm{CM}}=55.6$–77.8%, mean 66.7%). This stricter indicator counts only risk items where both methods produced substantially equivalent descriptions at the same location. The higher value in Sample 02 (77.8%) reflects the agent’s capacity to independently identify the same core hazards as the manual assessor in a complex setting; the lower value in Sample 04 (55.6%) reflects a combination of the emergency-device gap and finer-grained differences in risk attribution for shared hazards.

##### Miss Rate

($R_{\mathrm{MO}}=0.0$–22.2%, mean 11.1%). The miss rate quantifies the proportion of manual reference items that the agent failed to capture. The zero miss rate in Sample 02 is notable given the high spatial complexity of this sample. The 22.2% miss rate in Sample 04 is entirely constituted by the two aforementioned emergency-call-device items, confirming that the framework’s primary blind spot lies in non-visual, equipment-inventory-type risks rather than in spatial or geometric hazards.

##### Precision

(P=45.0–70.0%, mean 57.5%). Precision measures the proportion of agent-generated items that correspond (completely or partially) to the manual reference. The lower precision in Sample 02 (45.0%) reflects the agent’s tendency to generate a substantially larger number of risk items than the manual assessor (20 vs. 9), with 11 agent-only items. Inspection of these 11 agent-only items reveals that the majority represent finer-grained decompositions of hazards that the manual assessor had noted only at a summary level, rather than spurious false alarms. The higher precision in Sample 04 (70.0%) reflects the closer convergence of the two methods in a structured environment where risk loci are more concentrated. We caution that precision values computed in this manner are sensitive to output granularity: an agent that systematically generates more fine-grained items will mechanically register lower precision against a coarser-grained reference, even when its additional items are substantively reasonable.

These indicators are computed against a single-assessor manual reference and primarily characterize the pattern of agreement and divergence rather than serving as validated accuracy metrics; their absolute values would shift under a different assessor or a multi-assessor consensus reference.

#### 3.3.4. Judgment Closure Analysis

Beyond comparison with the manual reference, an intrinsic performance dimension of the framework is its capacity to autonomously close risk judgments without requiring supplementary human measurement or verification. A risk item is classified as closed when the evidence chain assembled by the agent—combining geometric measurements, semantic observations, image evidence, and regulatory knowledge references—is sufficient to reach a definitive judgment; it is classified as open when one or more evidence components are insufficient, and the item is retained as an evidence gap pending further verification. Table 3 reports judgment closure statistics across the four pilot samples assessed under the standard protocol.

The mean closure rate of 71.7% indicates that, on average, approximately 28% of risk items could not be autonomously resolved by the agent and were retained as open evidence gaps requiring human follow-up. The open items were predominantly concentrated in two categories: (a) items requiring precise geometric measurements of object heights, clearances, or passage widths where the available keyframes provided insufficient observation angles or where the target features were partially occluded; and (b) items involving regulatory thresholds that required confirmation of non-visual attributes (e.g., material slipperiness, structural load capacity). This pattern identifies geometric measurement capability—specifically, the ability to autonomously trigger and complete metric measurements under suboptimal viewing conditions—as the primary bottleneck limiting judgment closure. Sample 01 (new apartment) achieved the highest closure rate (83.3%), consistent with the expectation that spaces with clear boundaries, modern fixtures, and low semantic ambiguity provide more favorable conditions for autonomous evidence assembly.

#### 3.3.5. Spatial Granularity Enhancement

A consistent pattern observed across both comparison samples was a systematic difference in the spatial granularity of outputs. Table 4 quantifies this difference.

The granularity enhancement factor is defined as $N_{\mathrm{Agent}}/N_{\mathrm{Manual}}$, measuring how many agent-generated risk items correspond to each manually identified risk area. In the spatially complex rural dwelling (Sample 02), the agent achieved a 2.2× enhancement, decomposing broad manual notations into independently addressable sub-items that can each serve as a discrete target for remedial action. In the structured urban apartment (Sample 04), the enhancement was modest (1.1×), with the agent’s added value manifesting primarily in measurement-supported verification rather than in further decomposition. This pattern suggests a dual-mode value proposition: the framework provides finer-grained local decomposition in spatially complex, ambiguous environments, and enhanced quantitative precision and evidence traceability in well-defined, structured environments.

#### 3.3.6. Geometric Measurement Demonstration

The framework’s geometric measurement capability was exercised in a targeted follow-up query for Sample 04. When prompted to verify doorway width at the entrance, the agent selected a suitable keyframe, generated OpenCV-based feature detection code to locate the door jambs in the 2D image, mapped the detected 2D pixel coordinates to 3D world coordinates via the frame’s canonical point map $X_{\mathrm{canon}}$ and camera pose $T_{wc}$, and computed the Euclidean distance, yielding a value of 1.216 m. The applicable threshold from GB 50763-2012 [20] (≥0.80 m for accessible doorways) was retrieved through the RAG tool, and the agent concluded that the doorway satisfied the regulatory minimum.

This measurement value has not been validated against an independent tape-measure ground truth. The reported 1.216 m represents the system’s internal geometric inference, whose accuracy is contingent on the AprilTag calibration chain, point cloud quality at the door jamb region, and the correctness of the LLM-generated OpenCV feature detection code. None of these error sources has been formally quantified; the demonstration is therefore intended to illustrate the measurement mechanism rather than to certify accuracy.

#### 3.3.7. Summary of Comparative Findings

Collectively, the pilot comparison yields the following indicative findings, each qualified by the limitations of the single-assessor reference design:

1. Risk coverage: The framework achieved recall of 77.8–100% against the manual reference, indicating that within its visual–spatial competence domain, it captures the majority of risk categories identified by a trained assessor. The two documented misses define a clear boundary condition for non-visual risk types.
2. Output granularity: The framework systematically produced finer-grained, object-level risk items (granularity enhancement of 1.1×–2.2×), decomposing room-level manual summaries into individually addressable sub-items. This enhancement was most pronounced in spatially complex environments (Sample 02, 2.2×).
3. Judgment autonomy: Approximately 71.7% of risk items were autonomously closed by the agent; the remaining ∼28% required human follow-up, predominantly for precise geometric measurements under suboptimal viewing conditions. Improving measurement autonomy is the single highest-leverage pathway to increasing overall system capability.
4. Measurement mechanism: The framework demonstrated a functional geometric measurement pipeline (query → frame selection → code generation → 2D-to-3D lifting → threshold comparison), but absolute measurement accuracy remains unvalidated against physical ground truth.
5. Validation maturity: All quantitative indicators reported here are computed against a single-assessor reference. Standard classification and agreement metrics (F1, Cohen’s κ), measurement error statistics (MAE, RMSE), and module-level ablation results are identified as priorities for the next validation phase.

## 4. Discussion

### 4.1. Interpretation of Pilot Results in Context

The pilot results reported in Section 3 must be interpreted within the constraints of the study design. The framework achieved risk identification coverage comparable to a single-assessor manual checklist in the two directly compared samples, but this pattern is not statistically generalizable. The apparent dual-mode advantage—finer-grained decomposition in complex environments (Sample 02) and enhanced measurement precision in structured environments (Sample 04)—may reflect the specific characteristics of these two dwellings rather than a stable, transferable property of the system. Without controlled variation in environmental complexity and without a larger sample, the context-dependency of the framework’s strengths remains a hypothesis.

From a methodological perspective, the Risk Prototype and SceneState architecture address a recognized gap between gerontological knowledge (typically expressed in narrative guidelines) and computational sensing (producing generic geometric and semantic data). The formalization of risk into five elements provides a reusable template for translating domain expertise into machine-verifiable criteria. The indicative performance indicators reported in Section 3.3—recall of 77.8–100% and precision of 45.0–70.0% against the manual reference—should be interpreted within the dual context of the framework’s methodological design and the pilot validation constraints. The recall-precision pattern (high recall with moderate precision in complex environments; convergent recall and precision in structured environments) is consistent with the dual-mode value proposition observed in Section 3.3.5: finer-grained decomposition in ambiguous settings versus measurement-supported verification in well-defined spaces. However, the value of the Risk Prototype formalization relative to simpler approaches (e.g., direct prompt engineering without structured evidence binding) has not been empirically isolated. The dual-pathway workflow—separating initial scanning from iterative verification—similarly lacks empirical validation: whether this separation improves overall assessment quality compared to a single-pass approach, or whether it introduces additional failure modes through multi-turn error accumulation, remains an open empirical question. The judgment closure analysis (mean closure rate of 71.7%, with open items concentrated in geometric measurement tasks) provides an empirical basis for prioritizing measurement autonomy as the primary target for methodological improvement. These uncertainties underscore the pilot status of the current validation and the need for more rigorous experimental designs in future work.

### 4.2. Limitations

The following limitations must be emphasized:

1. Validation scope: Five samples with manual comparison in two cases, using a single assessor without inter-rater reliability testing, cannot support claims of generalizability, statistical superiority, or clinical utility. The indicative recall, precision, and closure rates reported in Section 3.3 are computed against this single-assessor reference and would shift under a different assessor or a multi-assessor consensus standard. The results establish feasibility, not efficacy. The systematic difference in output granularity between agent and manual assessment (1.1×–2.2×) means that precision values are mechanically sensitive to granularity mismatch and should not be interpreted in isolation from the qualitative content of agent-only items.
2. Measurement accuracy: Metric values reported by the geometric measurement tool have not been validated against independent tape-measure ground truth. The AprilTag calibration chain lacks formal uncertainty quantification for corner detection error, viewing angle effects, and similarity transform assumptions.
3. Evidence imbalance: Visual observations currently dominate over geometric and knowledge-based evidence in the agent’s decision process. This creates risk of visually plausible but geometrically incorrect hypotheses being accepted.
4. Reproducibility: While key pipeline parameters are reported, full reproducibility would require release of the codebase and complete parameter documentation, which has not yet been accomplished.
5. AI Agent Robustness and Trustworthiness: The current pilot does not include dedicated experiments to verify the robustness and trustworthiness of the AI agent under adversarial or edge-case conditions. While we have implemented basic safeguards—such as sandboxed code execution for LLM-generated measurement scripts, confidence scoring for feature detections, and geometric plausibility checks for measurement outputs—these measures have not been formally validated against a benchmark of failure cases. The following remain unvalidated: (1) the agent’s behavior when SpatialLM produces systematic misclassifications; (2) the agent’s response when geometric measurements conflict with visual observations; (3) the stability of the LangGraph execution graph under prolonged multi-turn interactions; and (4) the containment guarantees of the sandboxed environment. Formal verification of agent behavior, including failure mode analysis and stress testing with corrupted or incomplete inputs, is required before deployment in safety-critical applications. These experiments are planned as part of future system hardening.
6. Computational cost: The framework’s runtime (approximately 10–28 min per dwelling for the initial scan, depending on video length and spatial complexity) was not systematically benchmarked against hardware configurations or optimized for deployment on resource-constrained devices. Claims of “low-cost” operation refer to hardware requirements (consumer smartphone) rather than computational efficiency.
7. **Relation to Prior Work**: This framework differs from prior AI-assisted building inspection studies in its explicit binding of multi-modal evidence to structured risk hypotheses. Unlike pure geometric analysis approaches (e.g., stair detection from point clouds) or pure semantic labeling systems, the Risk Prototype creates a verifiable bridge between sensed data and professional judgment criteria.
8. Quantitative Benchmarking Gap: This study does not include direct quantitative comparisons with alternative 3D reconstruction pipelines (e.g., NeRF-based methods, 3D Gaussian Splatting, or RGB-D SLAM systems) or with alternative indoor detection/segmentation frameworks (e.g., Mask R-CNN-, Segment Anything-, or PointNet++-based semantic segmentation). This omission is deliberate: our primary contribution is the orchestration and evidence-binding framework rather than algorithmic advances in individual components. A direct comparison of reconstruction or segmentation accuracy would not meaningfully capture the value of the Risk Prototype or SceneState. Nevertheless, we acknowledge that claims regarding overall end-to-end risk detection performance cannot be positioned relative to published state-of-the-art results. Systematic benchmarking on standard indoor datasets (e.g., ScanNet, Matterport3D) or on a dedicated residential safety benchmark would be required to establish comparative performance. Such benchmarking is a priority for future work.

## 5. Conclusions

This study presents a pilot implementation of a vision-based digital twin and AI agent framework for residential safety assessment, structured around the formalization of risk knowledge into computable Risk Prototypes and the organization of multi-modal evidence within a hierarchical SceneState data structure. By orchestrating existing open-source tools—MASt3R-SLAM for metric-scale reconstruction, SpatialLM for semantic parsing, and LangGraph for agent execution—around these evidence organization principles, the framework enables risk judgments to be grounded in geometric measurements, semantic labels, visual observations, and regulatory citations rather than relying solely on subjective interpretation.

Pilot validation across five heterogeneous residences, with detailed manual comparison in two representative cases, yielded the following indicative results: Against the single-assessor manual reference, the framework achieved risk recall rates of 77.8–100% and precision rates of 45.0–70.0%, with an average judgment closure rate of 71.7% across four samples under the standard protocol. In the spatially complex rural dwelling (Sample 02), the framework attained 100% recall (no risk category missed relative to the manual reference) with a granularity enhancement of 2.2×, decomposing room-level manual summaries into 20 object-specific, actionable risk items. In the structured urban apartment (Sample 04), recall was 77.8% (the two misses being items outside the framework’s visual evidence scope) with a precision of 70.0%, and measurement-supported verification of critical thresholds was demonstrated. These results suggest that the framework can achieve risk coverage comparable to manual checklist inspection in the tested cases while offering enhanced granularity in complex environments and quantitative precision in well-defined spaces. However, these findings must be interpreted with caution: all quantitative indicators are computed against a single-assessor reference and would shift under a different assessor or a multi-assessor consensus standard; the sample size is small (N = 5); no ground-truth tape measurements were collected for the independent validation of metric values; and standard statistical agreement metrics (e.g., Cohen’s κ) and classification metrics (e.g., F1-score) were not computed.

The primary contribution of this work lies in the task-specific orchestration and evidence organization principles that enable existing open-source tools to support safety-critical judgment: we demonstrate how narrative risk guidelines can be decomposed into structured, evidence-constrained templates (Risk Prototypes); how heterogeneous sensing data can be bound to these templates through a unified factual record (SceneState); and how an AI agent can conduct continuous, tool-augmented assessment through a dual-pathway workflow. This orchestration addresses the fragmentation between gerontological domain knowledge and computational perception, but it does not claim to replace human expertise or to constitute a clinically validated diagnostic tool.

Several limitations must be acknowledged and addressed in future work. First, the validation reported here is a pilot study; larger-scale evaluation with multiple assessors, ground-truth measurements, and statistical reliability testing is essential before any claims of generalizability or clinical utility can be made. Second, the geometric measurement tool, while operationally functional, lacks comprehensive validation against independently collected tape-measure data and formal verification of the LLM-generated code against annotated benchmarks. Third, the current implementation exhibits an evidence imbalance favoring visual observations over geometric and knowledge-based modalities, which may lead to the acceptance of visually plausible but geometrically incorrect hypotheses. Fourth, the computational runtime (approximately 10–28 min per dwelling for the initial scan on server-grade GPU hardware, with 3–8 min for MASt3R-SLAM reconstruction, 2–5 min for SpatialLM semantic parsing, and 5–15 min for agent-based assessment depending on the number of follow-up queries) has not been systematically optimized for resource-constrained deployment. All processing was conducted on a workstation with an NVIDIA RTX 4090 GPU and 64 GB RAM. Claims of “low-cost” operation refer exclusively to data capture hardware (consumer smartphone) rather than to computational efficiency or deployment cost. Real-time or on-device processing is not currently supported; cloud-based or edge-computing deployment strategies would be required for scalable community rollout.

Future work should proceed in three directions: First, the validation must be expanded to include larger and more diverse samples, multiple assessors with inter-rater reliability testing, and systematic collection of ground-truth tape measurements for all reported metric values. This is a prerequisite for any transition from pilot feasibility to practical deployment. Second, the evidence integration mechanism should be strengthened through explicit weighting schemes and conflict resolution protocols that balance visual, geometric, semantic, and knowledge-based evidence, reducing the current dominance of visual observations and improving robustness in weak-evidence regions. Third, the computational pipeline should be optimized for efficiency and packaged for deployment, including investigation of model quantization, edge computing options, and integration with community health or insurance-based preventive care workflows. Regarding the speculative direction of longitudinal monitoring, it is important to clarify that this does not imply unfeasible real-time continuous sensor monitoring across the entire residence. Instead, it is envisioned as a periodic reassessment process (e.g., conducted monthly or following a fall incident) using the same low-cost smartphone video scanning protocol to computationally compare successive SceneState records. However, both longitudinal monitoring and generative intervention planning, while conceptually aligned with the framework’s architecture, require substantial additional infrastructure and validation before they can be considered feasible extensions; we therefore treat them as long-term research horizons rather than immediate next steps. For the current implementation, we adopted the Qwen3-VL family of models (utilizing both the plus and flash variants) as our core reasoning engine. This choice was motivated by its state-of-the-art performance in vision-language tasks at the time of development, as well as its efficient cost-performance trade-off, allowing us to allocate the lighter flash model for routine queries and the more capable plus model for complex spatial reasoning and tool-calling scenarios. Future work will continuously evaluate newer, more capable models as they become available and can be seamlessly integrated into our established LangGraph-based orchestration framework.

## Figures and Tables

**Table 1:** Agent vs. Manual Assessment: Risk Item Matching Statistics.

Sample	Manual	Agent	CM	PM	MO	AO
02 (Rural)	9	20	7	2	0	11
04 (Urban)	9	10	5	2	2	3

**Table 2:** Derived Performance Indicators Computed Against the Manual Reference Standard.

Indicator	Symbol	Formula	Sample 02	Sample 04	Mean
Recall	*R*	$\left(N_{\mathrm{CM}}+N_{\mathrm{PM}}\right)/N_{\mathrm{Manual}}$	100.0%	77.8%	88.9%
Complete Match Rate	$R_{\mathrm{CM}}$	$N_{\mathrm{CM}}/N_{\mathrm{Manual}}$	77.8%	55.6%	66.7%
Miss Rate	$R_{\mathrm{MO}}$	$N_{\mathrm{MO}}/N_{\mathrm{Manual}}$	0.0%	22.2%	11.1%
Precision	*P*	$\left(N_{\mathrm{CM}}+N_{\mathrm{PM}}\right)/N_{\mathrm{Agent}}$	45.0%	70.0%	57.5%

*Note:* $N_{\mathrm{Manual}}$ and $N_{\mathrm{Agent}}$ denote the total number of risk items identified by the manual assessor and the agent, respectively. $N_{\mathrm{CM}}$, $N_{\mathrm{PM}}$, and $N_{\mathrm{MO}}$ denote the counts of complete matches, partial matches, and manual-only items.

**Table 3:** Judgment Closure Statistics Across Pilot Samples Under Standard Assessment Protocol.

Sample	Total Risk Items	Closed	Open	Closure Rate
01 (New apartment)	6	5	1	83.3%
02 (Rural dwelling)	20	14	6	70.0%
04 (Aged urban)	10	7	3	70.0%
05 (Aged urban)	11	7	4	63.6%
**Mean**	–	–	–	**71.7%**

Note: Sample 03 (early-2000s urban home, mobility-impaired elder) was included in system-level testing but is excluded from this table because its risk items were generated under an adaptive profiling mode (occupant-specific functional data were provided to the agent), making its closure conditions not directly comparable to those of the other four samples, which operated under the standard assessment protocol without occupant-specific functional priors.

**Table 4:** Spatial Granularity Comparison: Manual vs. Agent Outputs.

Dimension	Sample 02 (Rural)	Sample 04 (Urban)
Manual output granularity	Room-level summary (9 items)	Room-level summary (9 items)
Agent output granularity	Object-level localization (20 items)	Object-level localization (10 items)
Granularity enhancement factor	2.2×	1.1×

## Data Availability

The data presented in this study are available on request from the corresponding author. The data are not publicly available due to the privacy restrictions of the residential environments surveyed.
